# Epigenetic silencing of *JMJD5* promotes the proliferation of hepatocellular carcinoma cells by down-regulating the transcription of *CDKN1A*

**DOI:** 10.18632/oncotarget.6867

**Published:** 2016-01-09

**Authors:** Bing-Hao Wu, Hui Chen, Chun-Miao Cai, Jia-Zhu Fang, Chong-Chao Wu, Li-Yu Huang, Lan Wang, Ze-Guang Han

**Affiliations:** ^1^ Key Laboratory of Systems Biomedicine (Ministry of Education) and Collaborative Innovation Center of Systems Biomedicine of Rui-Jin Hospital, Shanghai Jiao Tong University School of Medicine, Shanghai, China; ^2^ Shanghai-MOST Key Laboratory for Disease and Health Genomics, Chinese National Human Genome Center at Shanghai, Shanghai, China; ^3^ Shanghai Center for Systems Biomedicine, Shanghai Jiao Tong University, Shanghai, China

**Keywords:** KDM8, tumorigenicity, cell cycle, transcription, histone modification

## Abstract

Proteins that contain jumonji C (JmjC) domains have recently been identified as major contributors to various malignant human cancers through epigenetic remodeling. However, the roles of these family members in the pathogenesis of hepatocellular carcinoma (HCC) are obscure. By mining public databases, we found that the HCC patients with lower JmjC domain-containing protein 5 (*JMJD5*) expression exhibited shorter survival time. We then confirmed that *JMJD5* expression was indeed decreased in HCC specimens, which was caused by the altered epigenetic histone modifications, the decreased H3K9ac, H3K27ac and H3K4me2/3 together with the increased trimethylation of H3K27 and H3K9 on the *JMJD5* promoter. Functional experiments revealed that *JMJD5* knockdown promoted HCC cell proliferation and *in vivo* tumorigenicity by accelerating the G1/S transition of the cell cycle; in contrast, ectopic *JMJD5* expression had the opposite effects. At molecular mechanism, we found that, in HCC cell lines including *TP53*-null Hep3B, *JMJD5* knockdown led to the down-regulation of *CDKN1A* and ectopic expression of JMJD5 not only increased but also rescued *CDKN1A* transcription. Moreover, *CDKN1A* knockdown could abrogate the effect of JMJD5 knockdown or overexpression on cell proliferation, suggesting that JMJD5 inhibits HCC cell proliferation mainly by activating *CDKN1A* expression. We further revealed that JMJD5 directly enhances *CDKN1A* transcription by binding to *CDKN1A*'s promoter independent of H3K36me2 demethylase activity. In short, we first prove that *JMJD5* is a tumor suppressor gene in HCC pathogenesis, and the epigenetic silencing of *JMJD5* promotes HCC cell proliferation by directly down-regulating *CDKN1A* transcription.

## INTRODUCTION

Compelling evidence demonstrates that altered chromatin remodeling plays an important role in tumorigenesis. Histone methylation is known to be regulated by histone methyltransferases and demethylases. LSD1/KDM1 was the first histone demethylase to be discovered; it can demethylate mono- and dimethylated lysine residues through a FAD-dependent amine oxidase reaction, and it has been considered an important contributor to some tumors [1, 2].

Recently, some members of jumonji C (JmjC) domain-containing protein family were identified as a second type of histone demethylase that can remove all methylation modifications on the lysine residues of histones [3, 4, 5]. All family members have a JmjC domain, which shares high similarity with the cupin metalloenzyme domain [6]. However, in addition to the subset of members with histone demethylase activity, many other members, including JMJD5, JMJD6 and TYW5, also possess protein or RNA hydroxylase activity [7, 8, 9]. Dysregulation of family members such as *JMJD5*, *JMJD6*, *JARID1B*, *JARID2*, *PHF8* and *JMJD2A* causes abnormal embryonic development or promotes cancer cell proliferation and migration [5, 10, 11].

It is unknown if JmjC domain-containing proteins are involved in the pathogenesis of hepatocellular carcinoma (HCC). To define which JmjC domain-containing proteins might contribute to HCC, we mined the gene expression profiles of all family members in HCC by analyzing public databases. Very interestingly, nineteen of the twenty-nine members of this family were misregulated in HCC specimens. Among them, the most significantly down-regulated gene is JmjC domain-containing protein 5 (*JMJD5*); nearly 80% of HCC specimens showed at least two-fold downregulation of this gene. Our evidence showed that reduced *JMJD5* promoted HCC cell proliferation and cell cycle progression by directly suppressing *CDKN1A* transcription. The present results provided novel insight into *JMJD5*'s function during HCC progression.

## RESULTS

### The expression patterns of JmjC family members show *JMJD5* downregulation in HCC specimens

**Figure 1:**
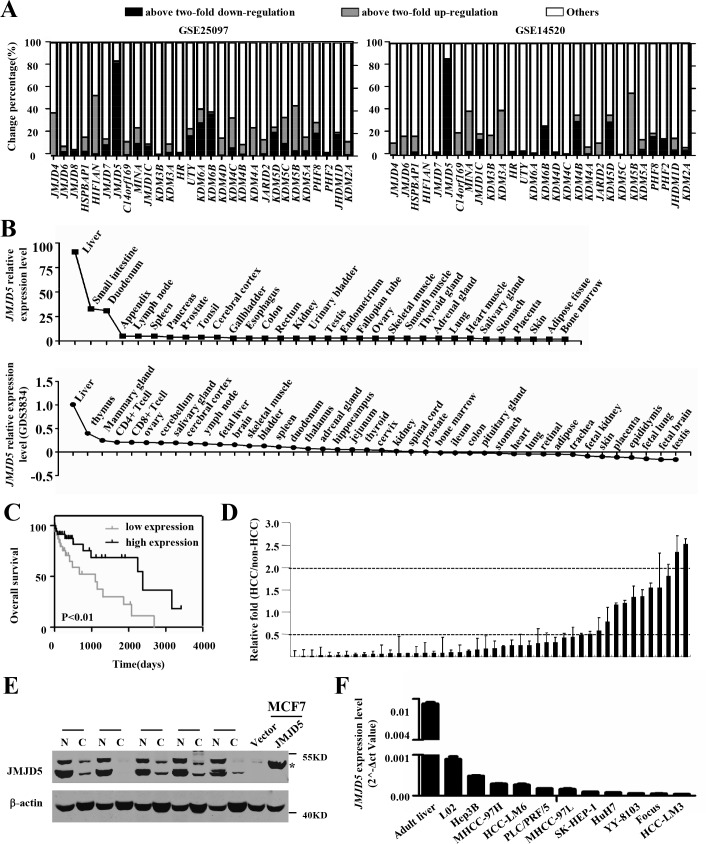
Tissue expression patterns and JMJD5 expression in HCC specimens and cell lines **A.** The expression patterns of JmjC family members in HCC tissue were analyzed based on two public datasets (GSE25097 and GSE14520). Each column represents one gene, and the *y*-axis indicates the percent change in gene expression. **B.** The relative *JMJD5* expression level in various human tissues and cells was evaluated based on THE HUMNA PROTEIN ATLAS and a public database (GDS3834). **C.** The result of Kaplan-Meier survival analysis exhibited that patients with low expression of *JMJD5* (*n* = 47) had shorter overall survival time than those with high *JMJD5* expression (*n* = 47). **D.** *JMJD5* expression was measured in 46 paired HCC and adjacent, non-cancerous livers using real-time RT-PCR, each column represented one paired sample, and y axis indicated the fold change. 0.5 and 2 meant that JMJD5 was down-regulated or up-regulated two fold in HCC. **E.** Five paired HCC and corresponding adjacent, non-cancerous liver samples were chosen to evaluate the expression of JMJD5 protein by Western blot assay, where MCF7 cells expressing ectopic JMJD5 were served as positive control, asterisk indicated the JMJD5 band. N, adjacent, non-cancerous livers; C, HCC samples. **F.** mRNA expression of *JMJD5* was detected in HCC cell lines and adult liver using real-time RT-PCR.

To explore if JmjC family members are involved in HCC pathogenesis, we first used public GEO datasets to evaluate the gene expression patterns of known JmjC family members in human HCC. Two databases (GSE25097 and GSE14520) were chosen because they contain comprehensive gene expression profiles for more than 100 paired HCC samples. Interestingly, a few JmjC family members exhibited similar expression patterns in at least 30% of the HCC samples in both datasets (Figure 1A and Supplemental Figure 1A). *KDM5B* was significantly upregulated at least two-fold in 40% of the HCC specimens, whereas *JMJD5* was significantly downregulated at least two-fold in 82% of the samples. *KDM5B*, also named *JARID1B* or *PLU-1*, has been considered an oncogene; it encodes a histone H3 lysine 4 (H3K4) demethylase and is localized on chromosome 1q32.1 [12, 13]. However, the function and mechanisms of action of *JMJD5*, also named *KDM8*, in tumorigenesis were relatively unclear. Although retroviral insertional mutagenesis originally identified *JMJD5* as a tumor suppressor gene [14], it has recently been reported that *JMJD5* overexpression promoted breast cancer cell proliferation [15]; this result suggests that *JMJD5* may suppress tumors or promote cancer in a cell context-dependent manner. The *JMJD5* expression pattern in HCC implied that *JMJD5* may function as a tumor suppressor in this cancer. To address this possibility, in the present work, we focused on the role and mechanism of action of *JMJD5* in HCC pathogenesis.

Using the public databases, GDS3834 and THE HUMAN PROTEIN ATLAS [16], we analyzed the *JMJD5* expression pattern in human tissue. Very interestingly, *JMJD5* was most highly expressed in the liver (Figure 1B), suggesting that *JMJD5* could play an important role in hepatic functions and physiology. By analyzing TCGA data we surprisingly found that the lower expression of *JMJD5* was significantly correlated with age (≤ 60 or > 60 year old, *P* = 0.0082), tumor stages (*P* < 0.05), overall survival (≤ 24 or > 24 months, *P* = 0.0222) and overall survival status (dead or alive, *P* = 0.034) (Table 1). But it was not associated with gender, metastasis stage and recurrence (*P* > 0.05) (Table 1). A Kaplan-Meier survival analysis exhibited that HCC patients with low *JMJD5* expression had shorter survival time compared with those with high *JMJD5* expression (*P* < 0.01) (Figure 1C), and the median survival of HCC patients with low *JMJD5* expression and high *JMJD5* expression was 987 and 2141 days respectively. Furthermore, we confirmed downregulation of *JMJD5* in HCC samples and cell lines. *JMJD5* was significantly downregulated in 29/46 (63%) samples, as shown by real-time RT-PCR (Figure 1D); in 43/63 (68.3%) different HCC samples, as shown by semi-quantitative PCR (Supplemental Figure 1B); as well as in five paired HCC samples, as shown by western blotting (Figure 1E). Moreover, compared with the normal adult liver, *JMJD5* was markedly downregulated in all examined HCC cell lines, as shown by real-time RT-PCR and semi-quantitative RT-PCR (Figure 1F and Supplemental Figure 1C). The data collectively indicated that *JMJD5* was significantly downregulated in most HCC samples.

**Table 1:** The correlations of JMJD5 expression with clinical characteristics of HCC

Clinical character	Clinical Groups	NO. of patients	JMJD5		χ^2^	P value
			Low^a^	High^b^		
***Age (Years)***	≤60	55	35	20	6.993	0.008
	>60	88	36	52		
***Gender*** Male	87	46	41	0.923	0.337
	Female	56	25	31		
***Metastasis stage***	M0	108	53	55	0.361	0.548
	M1	3	2	1		
***Recurrence***	Yes	16	8	8	0	1
	No	46	23	23		
***Tumor stage***	T1	58	21	37		
	T2	39	23	16	4.877	0.027
	T3	38	21	17	3.388	0.066
	T4	8	6	2	4.377	0.036
***Overall survival (month)***	≤24	91	51	40	5.233	0.022
	>24	31	10	21		
***Overall survival status***	Alive	63	25	38	4.476	0.034
	Dead	80	46	34		

a HCC patients were ranked according to JMJD5 expression from high to low, the bottom 50% were served as JMJD5 low group.

b The top 50% were defined as JMJD5 high group.

All data were collected from TCGA. Chi-square test was utilized to estimate the significant difference. P< 0.05 was considered to be significant.

### *JMJD5* downregulation in HCC is caused by an epigenetic mechanism

**Figure 2:**
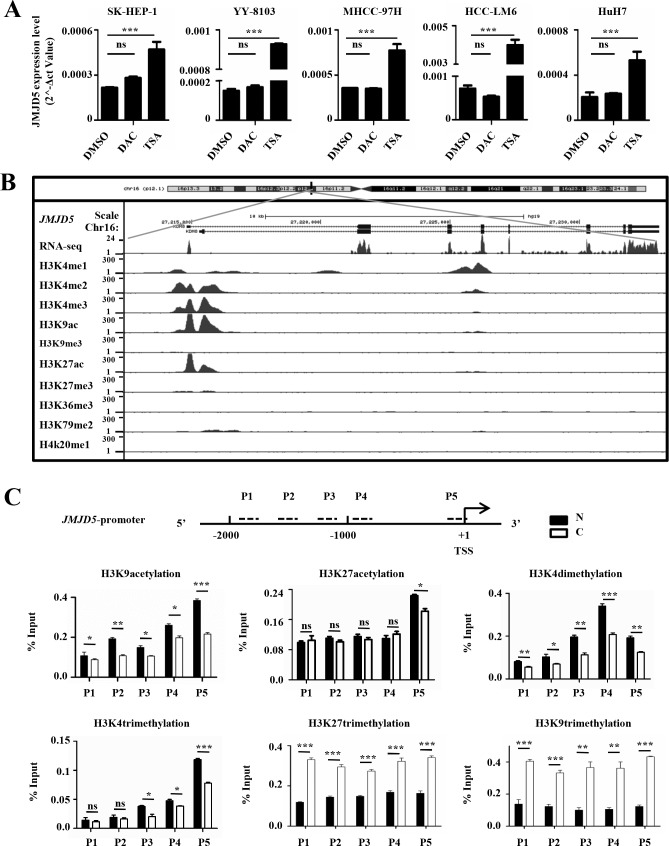
Epigenetic silencing of JMJD5 in HCC specimens and cells **A.** The *JMJD5* mRNA expression level was evaluated by real-time RT-PCR in five HCC cell lines after treatment with TSA or DAC. DMSO treatment served as a control. **B.** The histone modification patterns at the *JMJD5* locus were analyzed using UCSC ChIP-seq data from HepG2 cells. **C.** ChIP assays followed by real-time RT-PCR, were performed to evaluate the enrichment of histone modifications on the *JMJD5* promoter in HCC samples. The dotted lines indicate PCR primer (P1, P2, P3, P4 and P5) positions, and the numbers indicate the distance from transcription start site (TSS). *, *P <* 0.05; **, *P <* 0.01; ***, *P <* 0.001; ns, no significant difference.

To investigate why *JMJD5* was silenced in HCC, we used DAC, a DNA methyltransferase (DNMT) inhibitor, and TSA, a histone deacetylase (HDAC) inhibitor, to treat HCC cell lines. Interestingly, treatment with TSA, but not with DAC, could trigger *JMJD5* expression in SK-HEP-1, HuH-7, YY-8103, MHCC-97H and HCC-LM6 cells (Figure 2A and Supplemental Figure 2A), implying that, although the *JMJD5* promoter contains one CpG islands, it is histone modifications, not DNA methylation, that could be the major mechanism for regulating *JMJD5* transcription. And the bisulfite sequencing of the CpG islands in the *JMJD5* promoter also showed that there were no difference between the HCC specimens and the adjacent, non-cancerous liver tissue (data not shown). Next, we analyzed the histone modifications on the *JMJD5* locus using the public chromatin immunoprecipitation (ChIP)-seq datasets from HepG2 hepatic cells. The data showed that acetylation of lysine 9 and 27 (H3K9ac and H3K27ac) as well as dimethylation and trimethylation of lysine 4 (H3K4me3 and H3K4me2) in histone H3, which are associated with active gene expression [17, 18, 19, 20, 21], was enriched in the *JMJD5* promoter, whereas two known repressive histone modifications, H3K9 and H3K27 trimethylation (m3), were reduced in the same region (Figure 2B). To determine if these histone modifications in the region were altered during HCC tumorigenesis, we performed ChIP assays with anti-H3K9ac, anti-H3K27ac, anti-H3K4me2, anti-H3K4me3, anti-H3K9m3 and anti-H3K27m3 antibodies, and data showed that H3K9ac, H3K27ac, H3K4me2 and H3K4me3 were significantly decreased in HCC specimens; by contrast, both H3K9me3 and H3K27me3 were significantly enriched in HCC compared with the adjacent, non-cancerous liver tissue (Figure 2C and Supplemental Figure 2B). These collective data indicated that downregulation of *JMJD5* in HCC may be primarily attributed to epigenetic regulation.

### *JMJD5* knockdown promotes HCC cell growth and tumorigenicity

**Figure 3:**
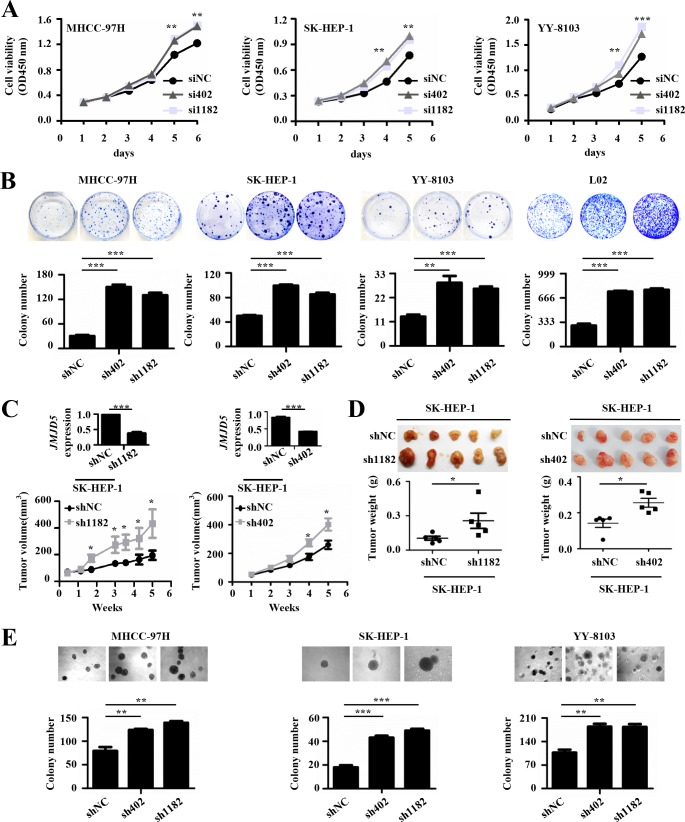
The effects of JMJD5 knockdown on the proliferation of HCC cells **A.** Three HCC cell lines were transfected with siRNAs against *JMJD5* (si402 and si1182), and cell viability was measured every day for 5 or 6 days to determine the cell growth curve. siNC served as a negative control. **B.** Four cell lines were transfected with plasmids containing shRNAs against *JMJD5* (sh402 and sh1182), and colonies were selected with G418. Plate colony formation is shown with representative plates (upper), and colony numbers were statistically analyzed (lower). **C.-D.** *In vivo* tumorigenicity of SK-hep1 cells with *JMJD5* stably knocked down. **C.** Tumor volumes were measured at the indicated time points. The *JMJD5* expression level in these cells is indicated at the top. Each group contained five mice. **D.** Tumors were removed (upper), weighed and statistically analyzed (lower). **E.** Three HCC cell lines were transfected with shRNAs against *JMJD5*, and colony formation on soft agar medium was analyzed. Soft agar colony formation is shown with representative dishes (top), and the number of colonies was statistically analyzed (bottom). All experiments were repeated at least three times. *, *P <* 0.05; **, *P <* 0.01; ***, *P <* 0.001.

Abnormal proliferation is one property of malignant tumors [22]. To evaluate the effect of *JMJD5* knockdown on HCC cell proliferation, we transiently transfected chemically synthesized small interfering RNAs (siRNAs) against *JMJD5* into HCC cell lines, including MHCC-97H, SK-HEP-1 and YY-8103, and found that siRNA-mediated *JMJD5* silencing significantly promoted the growth of these cells (Figure 3A). Subsequently we constructed a recombinant pSUPER plasmid encoding a short hairpin RNA (shRNA) against *JMJD5* and then transfected it into MHCC-97H, SK-HEP-1, YY-8103, L02 and Hep3B cells. Interestingly, *JMJD5* knockdown also significantly promoted colony formation in these cells (Figure 3B and Supplemental Figure 3A). Furthermore, SK-HEP-1 and YY-8103 cells containing a stable shRNA against endogenous *JMJD5* were subcutaneously inoculated into the flanks of nude mice; as a control, equal quantities of cells containing the negative control shRNA were injected into the opposite flank of the same mice. Significantly, shRNA-mediated *JMJD5* knockdown promoted the *in vivo* tumorigenicity of the HCC cells, as shown by their increased xenograft tumor size and weight compared with that of the cells with the empty vector (Figure 3C, 3D and Supplemental Figure 3B-3D). We also performed soft agar colony formation assays to investigate the anchorage-independent growth. The results showed that knockdown of endogenous *JMJD5* clearly promoted the anchorage-independent growth of MHCC-97H, SK-HEP-1 and YY-8103 cells (Figure 3E). These data suggested that reduced *JMJD5* expression may facilitate the tumorigenesis and progression of HCC.

### *JMJD5* overexpression inhibits HCC cell growth and tumorigenicity

**Figure 4:**
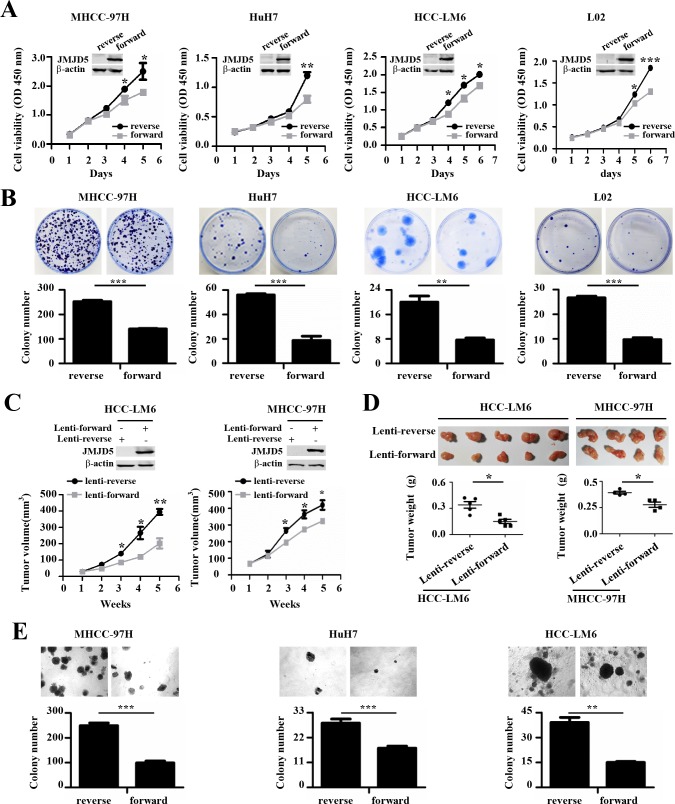
The anti-proliferative role of JMJD5 in HCC cells **A.-B.** The proliferation of four hepatic cell lines transfected with plasmids expressing JMJD5 (forward) was evaluated. **A.** Cell viability was calculated every day for 5 or 6 days to determine the cell growth curve. A plasmid containing the reverse *JMJD5* sequence was used as a control, and JMJD5 protein expression, as indicated by western blot, is shown at the top. **B.** Plate colony formation is shown with representative dishes (top) and the number of colonies was statistically analyzed (bottom). **C.-D.** *In vivo* tumorigenicity of MHCC-97H and HCC-LM6 HCC cells stably expressing JMJD5. **C.** Tumor volumes were measured at the indicated time points. The JMJD5 expression level in these cells is indicated at the top. Each group contained four or five mice. **D.** Tumors were removed (upper), weighed, and then statistically analyzed (lower). **E.** Three HCC cell lines were transfected with JMJD5, and colony formation in soft agar medium was analyzed. All experiments were repeated at least three times. *, *P <* 0.05; **, *P <* 0.01; ***, *P <* 0.001.

To further explore the effect of *JMJD5* on HCC cell proliferation, we transiently transfected the recombinant plasmid pcDNA3.1B, which encodes JMJD5, into MHCC-97H, HuH-7, HCC-LM6 and L02; as a negative control, a plasmid containing the reverse *JMJD5* sequence was used. As expected, compared with the controls, ectopic *JMJD5* expression significantly inhibited the growth and colony formation of these cells (Figure 4A, 4B). Furthermore, ectopic *JMJD5* expression significantly suppressed the *in vivo* tumorigenicity of HCC-LM6 and MHCC-97H cells after they were subcutaneously injected into the flanks of nude mice, as shown by the reduced size and weight of xenograft tumors (Figure 4C, 4D and Supplemental Figure 4A). *JMJD5* overexpression also reduced the anchorage-independent colony formation of MHCC-97H, HuH-7, HCC-LM6 and HCC-LM3 cells (Figure 4E and Supplemental Figure 4B). The above results collectively implied that *JMJD5* may function as a tumor suppressor in HCC cells by suppressing cell proliferation.

### JMJD5 regulates the G1-S transition of the cell cycle

**Figure 5:**
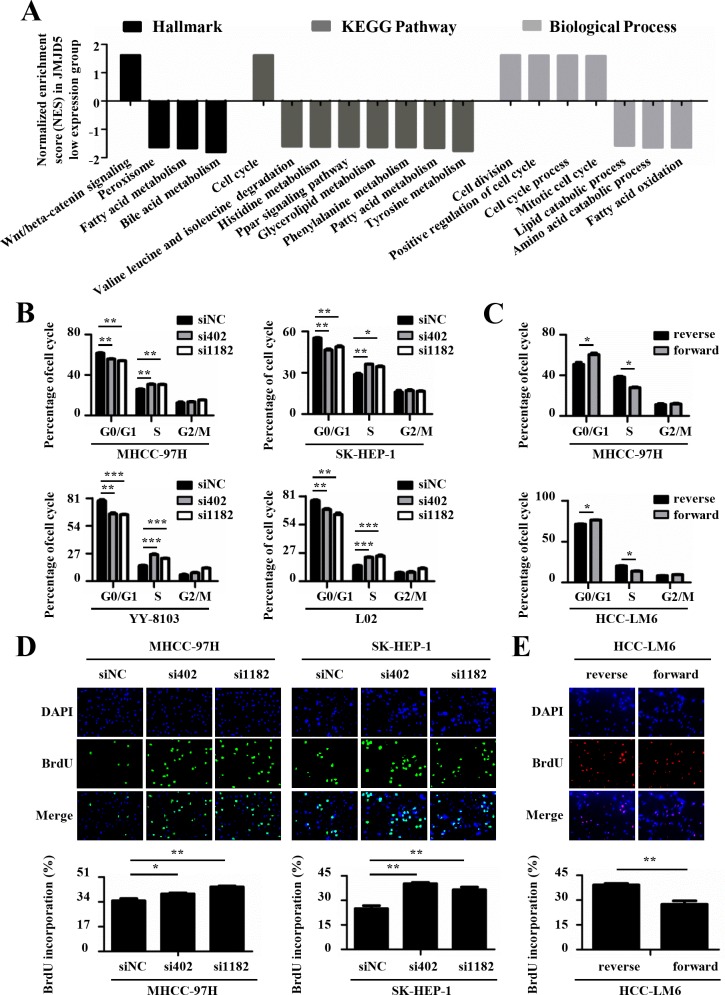
JMJD5 suppresses the cell cycle progression of HCC cells **A.** Public dataset (GSE14520) was divided into two groups, higher JMJD5 expression group and lower JMJD5 expression group, and the normalized enrichment score (NES) of three gene set categories was calculated by Gene Set Enrichment Analysis (GSEA). Each column represented one statistically significant gene set (*P* < 0.05). The positive and negative NES represented these up-regulated and down-regulated gene sets in the lower JMJD5 expression group, respectively. **B.** Cell cycle analysis of HCC cells transfected with siRNAs against *JMJD5*, and **C.** cell cycle analysis of HCC cells ectopically expressing JMJD5. The percentages of the cell subpopulations at different stages of the cell cycle were statistically analyzed. **D.-E.** Immunofluorescence images (200x) showing HCC cells with BrdU incorporation after *JMJD5* knockdown **D.** or overexpression **E.**, where the percentages of cells with BrdU incorporation were statistically analyzed (bottom). All experiments were repeated at least three times. *, *P <* 0.05; **, *P <* 0.01; ***, *P <* 0.001.

To explore the mechanism by which *JMJD5* regulates cell proliferation, we carried out Gene Set Enrichment Analysis (GSEA) using public datasets (GSE14520), the results showed that lower *JMJD5* expression was positively associated with Wnt/β-catenin pathway which is well-known to be activated during HCC development [23, 24], and cell cycle progress (Figure 5A and Supplemental Figure 5A). In addition, we also found that lower JMJD5 expression was negatively associated with metabolism processes such as lipid and amino acid catabolism (Figure 5A). Subsequently, we used FACS to assess the effect of *JMJD5* knockdown or overexpression on cell cycle progression. Interestingly, siRNA-mediated *JMJD5* knockdown significantly promoted the G1-S transition of MHCC-97H, SK-HEP-1, YY-8103 and L02 cells, whereas ectopic *JMJD5* expression delayed the G1-S transition in MHCC-97H and HCC-LM6 cells (Figure 5B, 5C and Supplemental Figure 5B, 5C). This suggested that *JMJD5* may regulate cell cycle progression in HCC cells. To further test this hypothesis, we performed 5-bromo-2′-deoxyuridine (BrdU) incorporation assays to assess the effect of *JMJD5* knockdown or overexpression on newly synthesized DNA during the cell cycle. The result showed that siRNA-mediated *JMJD5* knockdown indeed led to an increased proportion of cells in S phase, as indicated by BrdU incorporation in MHCC-97H, SK-HEP1, YY-8103 and L02 cells; by contrast, ectopic *JMJD5* expression reduced BrdU incorporation in HCC-LM6 cells (Figure 5D, 5E and Supplemental Figure 5D). Collectively, these observations suggested that *JMJD5* may influence the G1-S transition in HCC cells.

### JMJD5 affects HCC cell proliferation by regulating *CDKN1A* transcription

**Figure 6:**
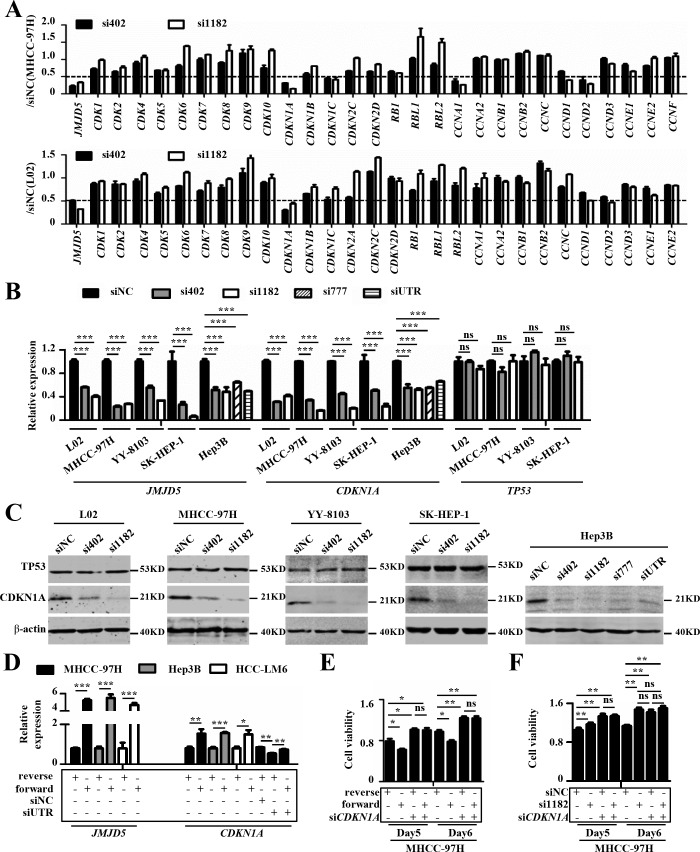
JMJD5 influences HCC cell proliferation primarily by regulating CDKN1A transcription **A.** The expression of cell cycle-related regulators were performed using real-time RT-PCR in MHCC-97H and L02 cells after *JMJD5* knockdown. The y-axis indicates the ratio of the mRNA levels of the given genes after siRNA-mediated *JMJD5* knockdown to that of siNC. **B.** Real-time RT-PCR was used to measure *CDKN1A* and *TP53* mRNA levels in HCC cell lines with siRNA-mediated *JMJD5* knockdown. **C.** Five cell lines were transfected with siRNAs against *JMJD5* and the levels of CDKN1A and TP53 proteins were tested by western blot. **D.** Ectopic JMJD5 expression increased *CDKN1A* mRNA expression and rescued the decrease in *CDKN1A* mRNA mediated by *JMJD5* knockdown. **E.** CDKN1A knockdown abrogated the decreased cell viability mediated by *JMJD5* overexpression. **F.** *CDKN1A* knockdown also abrogated the increased cell viability induced by *JMJD5* knockdown. All experiments were repeated at least three times. *, *P <* 0.05; **, *P <* 0.01; ***, *P <* 0.001; *ns*, no significant difference.

It is well-known that cell cycle progression is regulated by cyclins, cyclin-dependent kinases (CDKs), and cyclin-dependent kinase inhibitors [25, 26]. To clarify the molecular mechanism by which *JMJD5* regulates the G1-S transition in HCC cells, we used real-time RT-PCR to analyze the mRNA levels of known molecules involved in the G1-S transition, including *RB, CDKN1A, CDK2* and *CCNE*, in MHCC-97H and L02 cells that were silenced for JMJD5. Interestingly, only the mRNA level of *CDKN1A*, a well-known negative regulator of the G1-S transition, was significantly reduced at least two-fold in both cell lines after *JMJD5* knockdown (Figure 6A). We then determined the expression levels of *CDKN1A* and its well-known upstream transcription factor, *TP53*, in L02, MHCC-97H, SK-HEP-1, YY-8103 and the *TP53*-null Hep3B cells after *JMJD5* silencing. We found that siRNA-mediated *JMJD5* knockdown dramatically reduced the mRNA and protein levels of *CDKN1A*, but not *TP53* (Figure 6B, 6C). To further confirm the finding, we next determined the *CDKN1A* premature mRNA (pre-mRNA) levels in MHCC-97H, YY-8103, SK-HEP-1 and Hep3B cells after *JMJD5* knockdown, and we found that they were also significantly reduced (Supplemental Figure 6A, 6B). Moreover, we observed that *JMJD5* overexpression elevated *CDKN1A* mRNA levels in MHCC-97H, Hep3B and HCC-LM6 cells, and it even rescued the *JMJD5* knockdown-mediated silencing of *CDKN1A* in MHCC-97H cells (Figure 6D and Supplemental Figure 6C, 6D). These data indicated that JMJD5 may directly activate *CDKN1A* transcription in a *TP53*-independent manner.

To further verify the notion that JMJD5 suppresses HCC cell proliferation mainly by activating CDKN1A, we transfected siRNA against *CDKN1A* into HCC cells with *JMJD5* knockdown or overexpression. The results showed that CDKN1A knockdown could abrogate the effect of JMJD5 knockdown and overexpression on HCC cell proliferation (Figure 6E, 6F and Supplemental Figure 6E, 6F).

### JMJD5 regulates *CDKN1A* transcription by directly binding to the *CDKN1A* promoter in HCC cells

It has recently been reported that JMJD5 activates or inhibits transcription of downstream genes through binding to their promoters or gene body [15, 27, 28, 29]. Interestingly, the decreased JMJD5 expression could elevate *Cdkn1a* transcription through increasing H3K36me2 on the gene body in mouse embryonic fibroblast cells (MEF) [28], which appear to contrast to this study.

**Figure 7:**
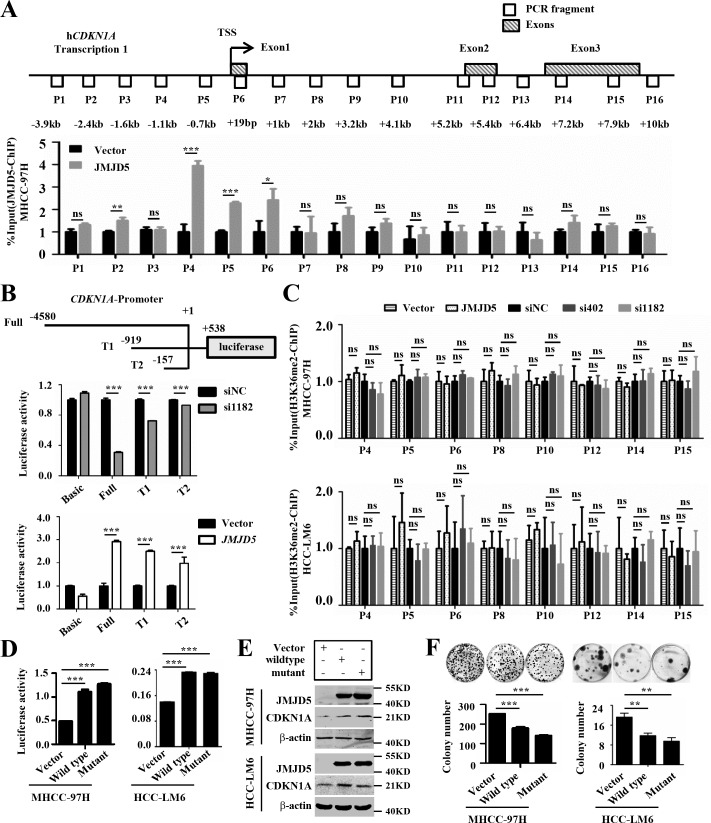
JMJD5 activates CDKN1A transcription by binding the CDKN1A promoter **A.** MHCC-97H cells were transfected with plasmids encoding JMJD5, and then anti-JMJD5 antibody was utilized to conduct ChIP assay. JMJD5 enrichment on *CDKN1A* gene locus was measured by real-time quantitative PCR, the blank boxes represent the genomic positions of these primers for PCR, the shadowy boxes indicate the exons based on the transcript variant 1 (Transcription 1), and the numbers show the distance from the primers to transcription start site (TSS). **B.** Recombinant luciferase reporter plasmids (Full, T1 and T2) with three different upstream fragments of the *CDKN1A* regulatory element (upper) were employed to evaluate the effect of *JMJD5* knockdown (middle) or overexpression (bottom) on *CDKN1A* transcription in MHCC-97H cells. A luciferase reporter vector without a promoter sequence was used to determine basal luciferase activity. **C.** Anti-H3K36me2 antibody was employed to carry out ChIP-qPCR assay, when JMJD5 knocked down and overexpressed in MHCC-97H and HCC-LM6 cells. **D.** A luciferase reporter system containing the upstream regulatory element of *CDKN1A* (Full) was used to evaluate the effect of the JMJD5^H321A^ mutant on *CDKN1A* transcription in both MHCC-97H and HCC-LM6 cells. **E.** Both wild type JMJD5 and JMJD5^H321A^ mutant increased CDKN1A protein expression in MHCC-97H and HCC-LM6 cells. **F.** JMJD5^H321A^ mutant inhibited the colony formation of both MHCC-97H and HCC-LM6 cells. All experiments were repeated at least three times. *, *P <* 0.05; **, P < 0.01; ***, P < 0.001; *ns*, no significant difference.

In order to clarify the conditions for the differential *CDKN1A* expression in HCC cells, we first detect whether JMJD5 could activate *CDKN1A* transcription by directly binding to *CDKN1A* gene locus in HCC cells, we transfected recombinant plasmids encoding the full length JMJD5 into MHCC-97H cells, and then we conducted ChIP-PCR assay with anti-JMJD5 antibody. The results showed that JMJD5 binds to the *CDKN1A* promoter, not gene body (Figure 7A). To explore the effect of JMJD5 knockdown and overexpression on *CDKN1A* promoter activity, we constructed a reporter gene system consisting of a luciferase reporter under the control of the *CDKN1A* promoter. We found that *JMJD5* knockdown decreased luciferase activity; by contrast, *JMJD5* overexpression increased luciferase activity, and the intensity of luciferase activity was positively associated with the length of the *CDKN1A* promoter (Figure 7B).

In addition, a previous study suggested that, in breast cancer, JMJD5 is a H3K36me2 demethylase [15] (Supplemental Figure 7A, 7B). However, western blots on whole-cell lysates from MHCC-97H, SK-HEP-1 and Hep3B cells showed that *JMJD5* overexpression or knockdown had no obvious effect on H3K36me2 and other histone modifications (Supplemental Figure 7A, 7B), which is consistent with recent reports [7, 30, 31]. To further define whether JMJD5's potential demethylase activity could influence *CDKN1A* transcription, we employed ChIP-PCR assay to assess the alteration of histone modifications on the *CDKN1A* locus, the results exhibited that the H3K36me2 modification had no significant difference on *CDKN1A* promoter and gene body whatever JMJD5 knockdown and overexpression (Figure 7C). Subsequently, we constructed a JMJD5 mutant with a H321A mutation in the ferrous iron binding site, which is responsible for its enzymatic activity [15]. Similarly, the JMJD5 mutant not only increased *CDKN1A* expression and luciferase reporter activity, but it also inhibited the colony formation of MHCC-97H and HCC-LM6 cells (Figure 7D-7F). Subsequently we further identified the necessary domain of JMJD5 responsible for CDKN1A activation by using the constructed *JMJD5* deletion mutants, here we found that N-terminal amino acid residues (aa111-270), but not JmjC domain, of JMJD5 was required for CDKN1A activation (Supplemental Figure 8). Collectively our data suggested that JMJD5 could directly bind to the *CDKN1A* promoter, enhancing *CDKN1A* transcription in an enzyme activity-independent manner.

To further define the conditions for different *Cdkn1a* transcription in MEFs, we also analyzed the effect of Jmjd5 on *Cdkn1a* transcription. Our data exhibited that Jmjd5 knockdown increased *Cdkn1a* transcription and inhibited G1-S transition of the cell cycle (Supplemental Figure 9A, 9B), which were consistent with the published report [28]. However, in contrast to the data in HCC cells, Jmjd5 bond to *Cdkn1a* gene body, not its promoter (Supplemental Figure 9C). Interestingly, as *Jmjd5* knockdown, the H3K36me2 modification was increased on *Cdkn1a* gene body but not promoter (Supplemental Figure 9D). These collective data suggested that the opposite effect of JMJD5 on *CDKN1A* transcription in HCC and MEF cells could be ascribed to the binding regions of JMJD5. In HCC cells, JMJD5 may function as a co-activator *via* binding to the *CDKN1A* promoter, whereas, in MEFs, Jmjd5, binding to *Cdkn1a* gene body, could exert the histone demethylase activity that modifies H3K36 methylation and alters the downstream gene transcription.

In addition, as comparison of HCC cells examined in this study, we also evaluated the effect of JMJD5 on breast and lung cancer cells. The results showed that JMJD5 overexpression promoted the proliferation of MCF7 breast cancer cells and A549 lung cancer cells, whilst JMJD5 knockdown inhibited their proliferation (Supplemental Figure 10). These data indicated that JMJD5 plays tumor-suppressive or cancer-promoting roles in a cell context-dependent manner.

## DISCUSSION

Some members of the JmjC family have recently been identified as a second type of histone demethylase, and dysregulation of a few family members, such as *PLU-1*, *PHF8*, *JMJD3* and *UTX*, may contribute to various human diseases, including tumors, through epigenetic regulation [13, 32, 33]. However, the role of this family in HCC remains unclear.

*JMJD5* participates in different physiological and pathological processes, including osteoclastogenesis, embryonic development, circadian clock regulation, stem cell differentiation and cancer progression [7, 15, 27, 28, 34, 35]. Suzuki et al used insertional mutagenesis in Blm-deficient mice to identify *JMJD5* as a potential tumor suppressor that guaranteed genome integrity [14] and another group showed that *JMJD5* was necessary for accurate chromosome segregation [36]. These data suggested that *JMJD5* may function as a tumor suppressor in certain tumors. However, in lung cancer and breast cancer, JMJD5 functions as oncogene that promotes cancer cell proliferation [15, 37]. As well-known, many genes including some JmjC family members such as *KDM2B*, *KDM6A* and *KDM6B*, suppress or promote tumors in a cell context-dependent manner [38]. JMJD5 was reported to exert H3K36me2 demethylase activity or hydroxylase activity [7, 15]. In breast cancer, the H3K36me2 demethylase activity is essential for *CCNA1* transcription and cell proliferation, by contrast, in HCC, our data revealed that the enzyme activity is seemingly unnecessary for *CDKN1A* activation and cell proliferation (Figure 7D-7F). This suggested that the transcription of different genes triggered by JMJD5 could depend on distinct mechanisms including its enzyme activity in diverse cancers.

With regard to molecular mechanism, it has recently been reported that JMJD5 can activate or suppress gene expression at transcriptional level and post-translational level. At transcriptional level, JMJD5 functions as a co-activator to promote HIF-1α target genes and *miR302* transcription through binding to their promoters [27, 29], and also acts as the H3K36me2 demethylase to inhibit *CDKN1A* and *CCNA1* transcription *via* binding to their gene bodies [15, 28]. At post-translational level, JMJD5 as hydroxylase may accelerate NFATc1 degradation. In the present work, our data showed that JMJD5, acting as a transcriptional co-activator, directly binds to the promoter of *CDKN1A* to enhance its transcription (Figure 7A, 7B), which is not to consistent with the published reports in MEF cells and human stem cells [27, 28]. In MEF cells, Jmjd5 binds to *Cdkn1a* gene body and negatively regulates *Cdkn1a* transcription, depending on the H3K36me2 demethylase activity [28]. In human stem cells, JMJD5 is inclined to suppress *CDKN1A* expression at post-transcriptional level through microRNA [27]. However, in HCC, JMJD5 mainly binds to *CDKN1A* promoter (Figure 7A) and functions as a transcriptional cofactor to positively promote *CDKN1A* transcription independent on the demethylase activity (Figure 7B-7E). These results indicated that the different binding regions of JMJD5 to *CDKN1A* gene locus, which could associate with the H3K36me2 demethylase activity, and the regulation fashion, may lead to the opposite outcome of *CDKN1A* transcription in different cell types.

## MATERIALS AND METHODS

### Tissue specimens and cell lines

All specimens were harvested from patients who suffered from HCC and were informed consent. The liver tumor-derived cell lines SK-HEP-1, PLC/PRF/5, HuH-7, HepG2 and Hep3B were purchased from the Institute of Biochemistry and Cell Biology, Shanghai Institutes for Biological Sciences, Chinese Academy of Sciences. HCC cell lines MHCC-97H, MHCC-97L, HCC-LM3, HCC-LM6, YY-8103 and Focus were obtained from Zhong-Shan hospital, Fudan University. The immortalized human fetal liver cell line L02 was obtained from Shanghai Chang-Zheng Hospital, The Second Military Medical University. HCC cell lines including PLC/PRF/5, HuH-7, Hep3B, YY-8103, Focus and SK-HEP-1 were authenticated using short tandem repeats (STRs) sequencing and used within 6 months after authentication. All cells were permanently stocked at liquid nitrogen when obtained. The mouse embryonic fibroblast (MEF) cells were isolated from the 13.5 days of embryos.

### Antibodies and reagents

The following antibodies were used in this research: JMJD5 (ab106391) from Abcam; β-actin (sc-47778), BrdU (sc-32323), P53 (sc-6243), CDKN1A (sc53870) and Flag (sc-166355) from Santa Cruz Biotechnology; and histone modification-specific antibodies were purchased from Abcam and Millipore. Propidium iodide (P4864), bromodeoxyuridine (B9258), DAC (A3656) and TSA (T1952) were obtained from Sigma.

### Data mining of the gene expression profiles of JmjC family members

The expression patterns of JmjC family members in HCC specimens were analyzed based on public Gene Expression Omnibus (GEO) databases (GSE25097 and GSE14520). In brief, the ratios of expression in HCC *versus* in the adjacent normal liver specimens were calculated and log2 transformed. Genes with at least two-fold down-regulation or up-regulation were considered significant. The percentages of all samples with at least a two-fold change in gene expression were then calculated. The expression profile of JMJD5 in different human tissues and cells was analyzed in the GEO datasets (GDS: 3834) and THE HUMNA PROTEIN ATLAS (http://www.proteinatlas.org).

### Overall survival analysis

TCGA data were obtained from cBioPortal (http://www.cbioportal.org/index.do). 94 HCC patients with RNA-seq data and clinical information including last communication time and survival status were ranked according to *JMJD5* expression from high to low, the top 50% patients were defined as high expression group and the others were classified as low expression group. Kaplan-Meier survival analysis was utilized to draw the overall survival curve, and the statistical difference between two curve was calculated by log-rank test.

### 5-aza-2′-deoxycytidine (DAC) and trichostatin A (TSA) treatments

HCC cells were treated with 2 uM DAC for four days or with 300 ng/ml TSA for two days. The medium was refreshed every day, and RNA was isolated on the last day.

### Public histone modifications ChIP-seq data mining

Public histone modifications ChIP-seq data were obtained from UCSC and the UCSC Accessions were listed as follows: wgEncodeEH000096, wgEncodeEH000083, wgEncodeEH001024, wgEncodeEH000095, wgEncodeEH000082, wgEncodeEH000081, wgEncodeEH001023, wgEncodeEH000094, wgEncodeEH003087, wgEncodeEH001749, wgEncodeEH000127. The distribution of these histone modifications on JMJD5 gene locus was visualized with UCSC browser according to the manual instruction.

### Chromatin immunoprecipitation (ChIP) assay

ChIP assays were conducted using an EZ ChIP Kit (Millipore, Billerica, MA) according to the user manual.

### Cell proliferation

Cells were cultured in 96-well plates with 2000-3000 cells/well. To measure cell viability, a Cell Counting Kit-8 (CCK-8, Dojindo Laboratories, Kumamoto, Japan) was used according to the manufacturer's instructions.

### Construction of recombinant plasmids

Two short hairpin RNAs (shRNAs) against *JMJD5* were synthesized and inserted into the pSUPER vector (Oligoengine, Seattle, WA, USA), which was renamed sh402 and sh1182 based on their target sites. In addition, shRNA expressing irrelevant sequence served as a negative control and is referred to as shNC. To eliminate the effect of plasmid size on cell phenotype, the forward human *JMJD5* open reading frame (ORF) (forward) and reverse sequence (reverse) were obtained by RT-PCR and inserted into pcDNA3.1/myc-His(−)B-3 X FLAG-IRES-hrGFP, which was derived from pcDNATM3.1/myc-His(−)B (Invitrogen, USA). The *CDKN1A* promoter sequence was cloned from normal human liver cDNA and inserted into pGL3 vector. For lentivirus construction, the shRNA was inserted into the PLKO-1 vector, and the *JMJD5* forward and reverse sequences were inserted into the PCDH vector.

### Screening for stable cell lines

SK-HEP-1 cells were transfected with shNC, sh402 and sh1182, then selected on 0.8 mg/ml G418 (Life Technologies Inc., Grand Island, NY, USA) for 3 weeks. Clones derived from single cells were collected and evaluated for their knockdown efficiency. YY-8103 cells were transfected with lentiviruses expressing shNC or sh1182, and MHCC-97H and HCC-LM6 cells were separately transfected with lentiviruses expressing JMJD5 forward or reverse; subsequently, 2 μg/ml puromycin was used to screen for positive clones.

### Plate colony formation assays

Cells were seeded in triplicate in 35-mm dishes with 3000-5000 cells/dish. To kill non-transfected cells, 0.6-1 mg/ml G418 was applied three times a week. After 2-3 weeks, clones were stained with crystal violet and counted.

### *In vivo* tumor xenografts

Stable HCC cells were subcutaneously injected near the scapulas of 6-week-old nude mice. Once a tumor formed, its volume was measured at different time points using the formula: Volume = 0.5 X width^2^ X length. After 5-6 weeks, the mice were euthanized, and the tumors were dissected and weighed.

### Soft agar colony formation assays

Approximately 2000-3000 transfected cells were plated into 24-well plates containing 1% base agar and 0.5% top agar. After 2-3 weeks, the colonies were counted.

### Gene set enrichment analysis (GSEA)

Gene set enrichment analysis was carried out using GSEA software according to the references [39, 40]. For the relationship analysis between *JMJD5* expression level and three gene categories including Hallmark, KEGG Pathway and Biological Process, we firstly chose the top50 and bottom50 HCC tissues from the public database (GSE14520) according to *JMJD5* expression. Subsequently the higher *JMJD5* expression group, lower *JMJD5* expression group and gene sets were conducted for GSEA analysis.

### Cell cycle analysis

Cultured cells were collected using 1X trypsin and washed once in 1X PBS. The cells were resuspended in 1X PBS containing 0.2% Triton X-100, RNase A (100 μg/ml) and propidium iodide (50 μg/ml) and incubated for 30 min in the dark. The samples were subsequently measured using a FACSCalibur flow cytometer and CellQuest software (BD Biosciences, USA).

### BrdU incorporation assay

Cells were incubated with 100 μM BrdU (Sigma-Aldrich, USA) for 2 hours and fixed with paraformaldehyde. After perforation with 0.5% Triton X-100 and DNA denaturation with 2 mol/L HCl, the cells were incubated with anti-BrdU antibody to perform immunofluorescence. A fluorescence microscope was used to analyze the cellular incorporation of BrdU.

### Immunofluorescence assays

In brief, cells were fixed in paraformaldehyde at 4°C for 30 mins, then treated with 0.5% Triton X-100 at room temperature for 5 mins. Then, the cells were blocked with bovine serum albumin (BSA) and incubated with anti-BrdU antibody at room temperate for 2 hours, followed by incubation with AlexaFluor 488 (green)- or AlexaFluor 546 (red)-coupled secondary antibody for 1 hour.

### Dual -luciferase reporter assay

MHCC-97H and HCC-LM6 cells were co-transfected with a combination of pRL-TK, encoding *Renilla* luciferase, and pGL3, encoding luciferase fused with the *CDKN1A* promoter, together with an siRNA against *JMJD5* or a plasmid expressing *JMJD5*. Luciferase activity was measured 48 hours after transfection using the Dual-Luciferase Reporter Assay System Kit (Promega, Madison, WI, USA) according to the user manual.

### Statistics

Log-rank test was utilized to compare the survival curve, Chi-square test was used to compare the correlations of *JMJD5* expression with clinical characteristics of HCC, and Student's *t*-test was used to evaluate statistically significant differences of quantitative variables. *P* < 0.05 was considered significant.

## SUPPLEMENTARY MATERIAL FIGURES AND TABLES


