# Supplementary material for: Epigenetic silencing of JMJD5 promotes the proliferation of hepatocellular carcinoma cells by down-regulating the transcription of CDKN1A
Source: Oncotarget. 2016 Jan 9;7(6):6847–63. doi: 10.18632/oncotarget.6867 (PMC4872753; doi:10.18632/oncotarget.6867)
Supplement: Supplementary file 1 [file oncotarget-07-6847-s001.pdf]

Epigenetic silencing of *JMJD5* promotes the proliferation of hepatocellular carcinoma cells by down-regulating the transcription of *CDKN1A*

Supplementary Material

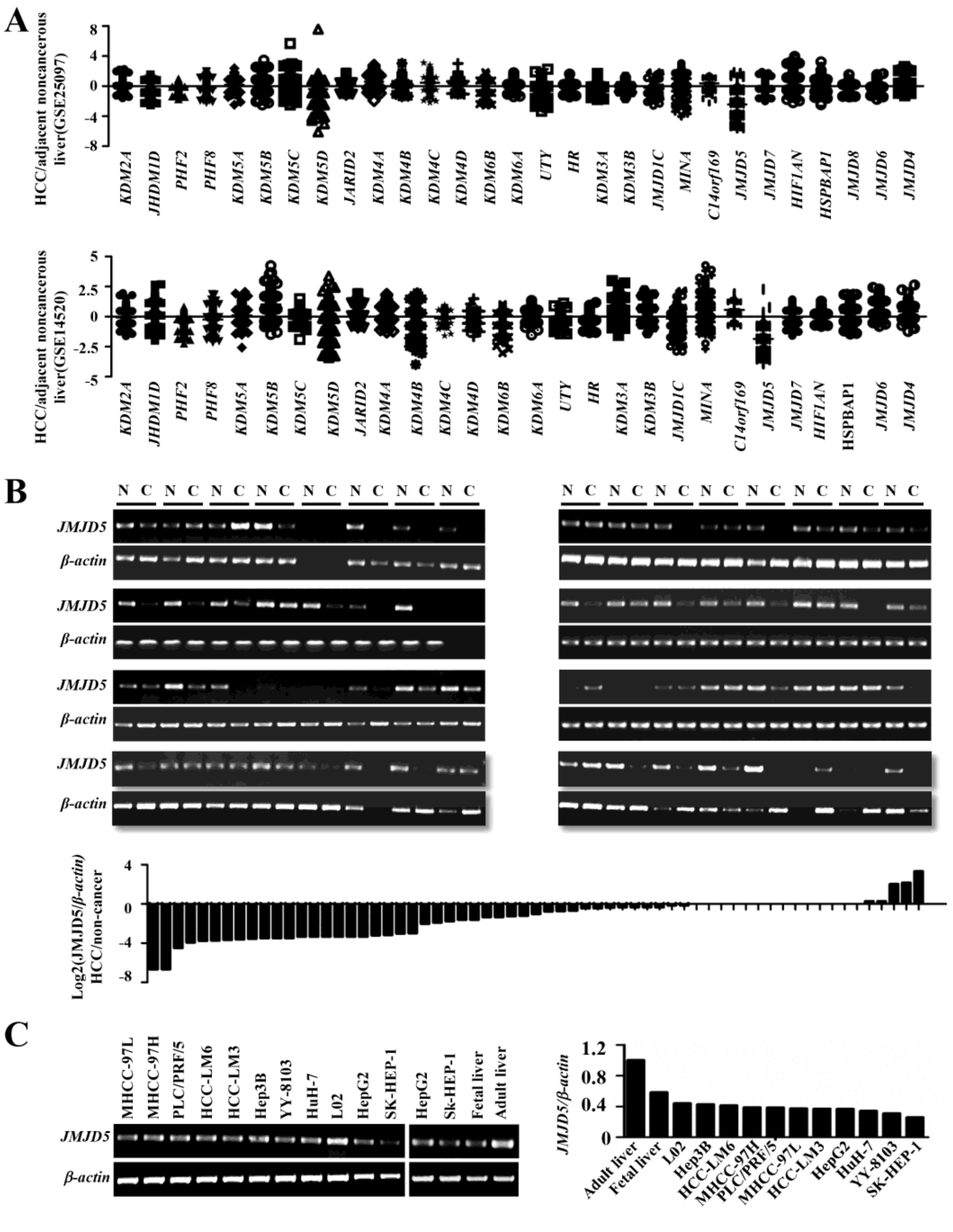

Supplemental Figure 1. The expression patterns of JmjC family members show

JMJD5 downregulation in HCC specimens. (A) The expression patterns of JmjC family members in HCC specimens were drawn based on two public databases (GSE25097 and GSE14520). The y-axis indicates the ratio of the mRNA level of a given gene in HCC to that in the adjacent, non-cancerous liver tissue. + and - represent up-regulation or down-regulation, respectively, of a gene in HCC compared with the adjacent, non-cancerous liver tissue. **(B)** A total of 63 paired HCC and adjacent, non-cancerous liver samples were used to detect the mRNA expression of *JMJD5* by RT-PCR (top). JMJD5 intensity was measured and normalized by β-actin, and the ratio of HCC and non-cancer liver was calculated and transformed by log2 (bottom). β-actin served as an internal control. N, adjacent, non-cancerous livers; C, HCC samples. **(C)** The expression of JMJD5 in HCC cell lines and normal liver was evaluated by semi-quantitative RT-PCR (left), and the relative quantification of bands was performed by gray scanning (right). The value of JMJD5/β-actin in adult liver was normalized 1. β-actin served as an internal control.

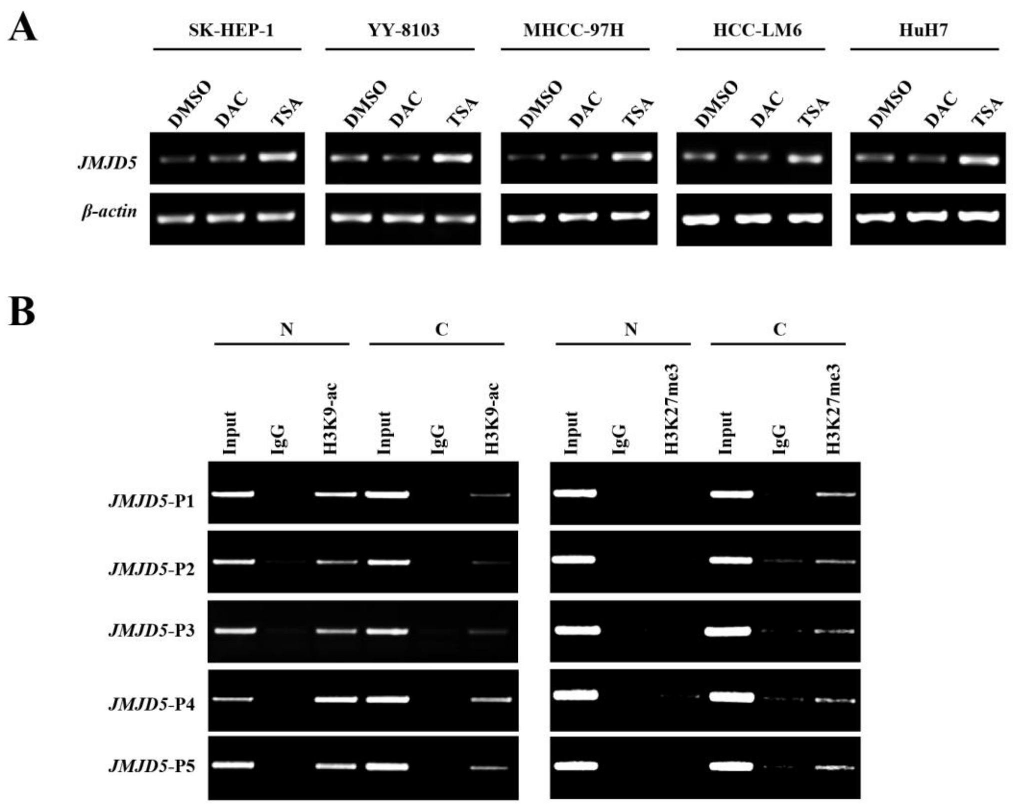

Supplemental Figure 2. JMJD5 downregulation in HCC is caused by an epigenetic mechanism. (A) RT-PCR was used to test the mRNA expression of *JMJD5* in five HCC cell lines after treatment with TSA or DAC. DMSO treatment served as a control, and β-actin was used as an internal control. **(B)** ChIP with anti-histone modification antibodies, followed by PCR, was used to detect enrichment for histone modifications (H3K9 acetylation and H3K27 trimethylation) on the *JMJD5* promoter in HCC samples. IgG served as a negative control. N, adjacent, non-cancerous livers; C, HCC samples.

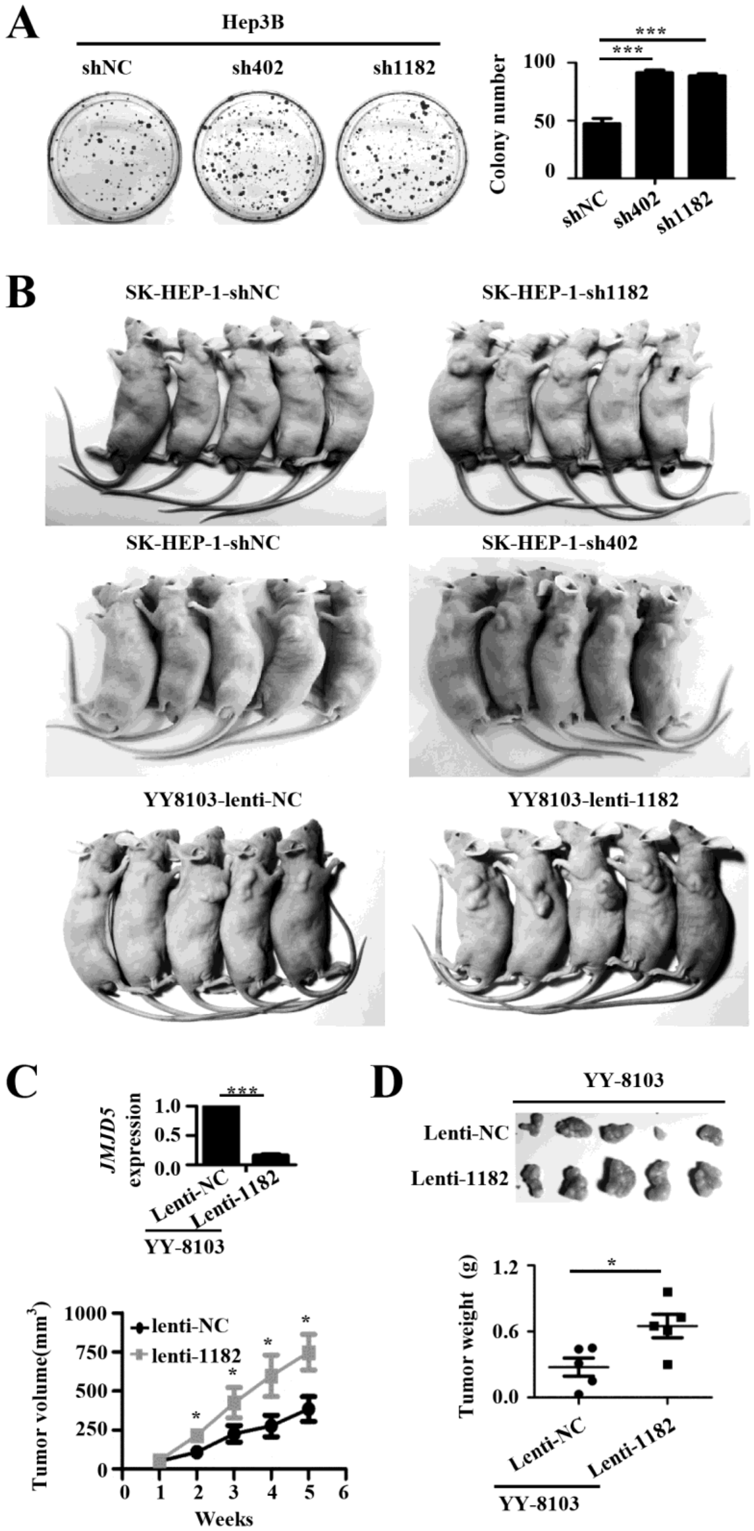

Supplemental Figure 3. JMJD5 knockdown promotes HCC cell growth and tumorigenicity. (A) Hep3B cells were transfected with plasmids containing shRNAs against *JMJD5* (sh402 and sh1182), and colonies were subsequently selected with

G418. Colony formation is shown with representative dishes (left), and the number of colonies was statistically analyzed (right). The experiment was repeated three times. **(B)** SK-hep1 and YY-8103 HCC cells with stable *JMJD5* knockdown were subcutaneously inoculated into nude mice, and mice with xenograft tumors are shown. **(C)** Tumor volume was measured for five weeks after the inoculation of YY-8103 cells expressing irrelative shRNA (lenti-NC) or shRNA against JMJD5 (lenti-1182), JMJD5 knockdown efficiency was detected by real-time qPCR (upper). **(D)** At 5 weeks, euthanasia was carried out, the tumour was removed (upper) and weighed (lower). Two-tailed t-tests were used to calculate significant differences. *, $P< 0.05$; ***, $P < 0.001$.

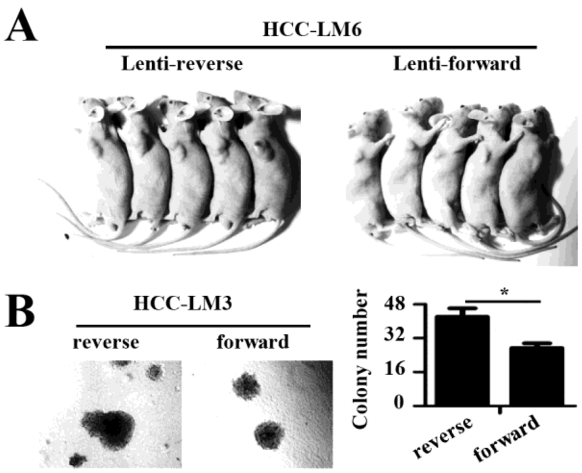

Supplemental Figure 4. JMJD5 overexpression inhibits HCC cell growth and tumorigenicity. (A) HCC-LM6 HCC cells stably expressing JMJD5 were subcutaneously inoculated into nude mice, and mice with xenograft tumors are shown. **(B)** HCC-LM3 HCC cells were transfected with *JMJD5*, and soft agar colony formation was analyzed. Soft agar colony formation is shown with representative dishes (left), and the colony numbers were statistically analyzed (right). All experiments were repeated at least three times, and a vector containing the reverse *JMJD5* sequence was used as a negative control. A two-tailed t-test was used to calculate significant differences. *, $P < 0.05$.

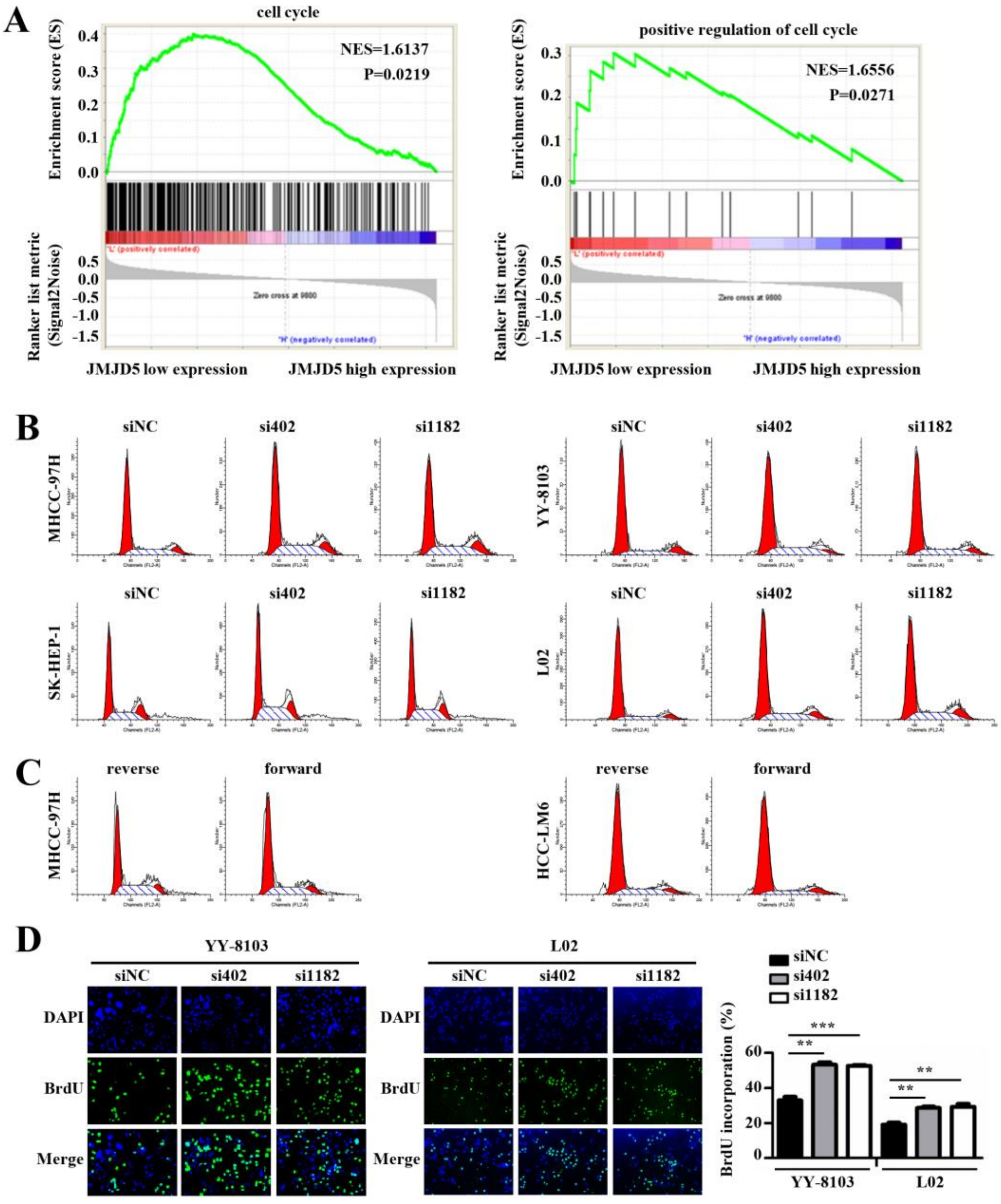

Supplemental Figure 5. JMJD5 regulates the G1-S transition of the cell cycle. (A) Gene set enrichment analysis was performed in HCC tissues with lower *JMJD5* expression versus higher *JMJD5* expression based on the gene sets of cell cycle progression. NES, normalized enrichment score. Flow cytometry was utilized to analyze the cell cycle of HCC cells transfected with siRNAs against *JMJD5* **(B)** or plasmids expressing ectopic JMJD5 **(C)**; irrelevant siNC or plasmids containing the reverse *JMJD5* sequence were used as negative controls. **(D)** Immunofluorescence

images showing YY-8103 (200X) and L02 (100X) cells with BrdU incorporation after *JMJD5* knockdown. The percentage of cells with BrdU incorporation was statistically analyzed (right). All experiments were repeated at least three times. **, P < 0.01; ***, P < 0.001.

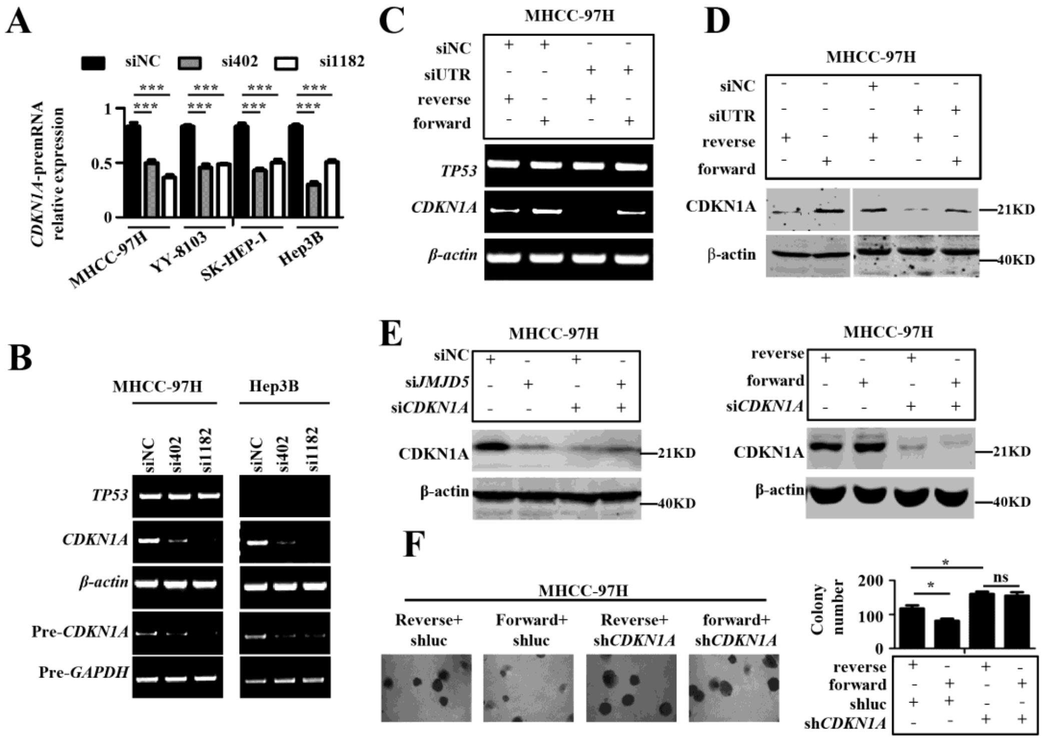

Supplemental Figure 6. JMJD5 affects HCC cell proliferation by regulating CDKN1A transcription. (A) *CDKN1A* premature mRNA was analyzed by real-time RT-PCR in MHCC-97H, YY-8103, SK-hep1 and Hep3B cells after JMJD5 knockdown. **(B)** MHCC-97H and Hep3B cells were transfected with siRNAs against *JMJD5*, and the expression of *CDKN1A* pre-mRNA (Pre- *CDKN1A*), as well as *CDKN1A* and *TP53* mRNA, was detected by RT-PCR. *GAPDH* pre-mRNA

(Pre-*GAPDH*) and *β-actin* were used as internal controls. **(C)** MHCC-97H cells were co-transfected with siRNAs and plasmids encoding ectopic JMJD5, and then the mRNA expression of *CDKN1A* and *TP53* was evaluated by RT-PCR. siNC and a plasmid containing the reverse *JMJD5* sequence served as negative controls, and *β-actin* was used as an internal control. **(D)** Western blot showed that ectopic JMJD5 increased the CDKN1A protein level and also rescued the *JMJD5* knockdown-mediated decrease in CDKN1A protein. **(E)** MHCC-97H cells were transfected with siRNA against JMJD5 or plasmid expressing JMJD5 after CDKN1A knockdown, western blotting was utilized to detect CDKN1A expression. **(F)** MHCC-97H cells were co-transfected with shRNA against CDKN1A and plasmid expressing JMJD5, anchorage-independent growth was detected by soft agar colony formation. The representative dishes were shown (left) and colonies number was calculated (right). *, $P<0.05$; ***, $P < 0.001$; *ns*, no significant difference.

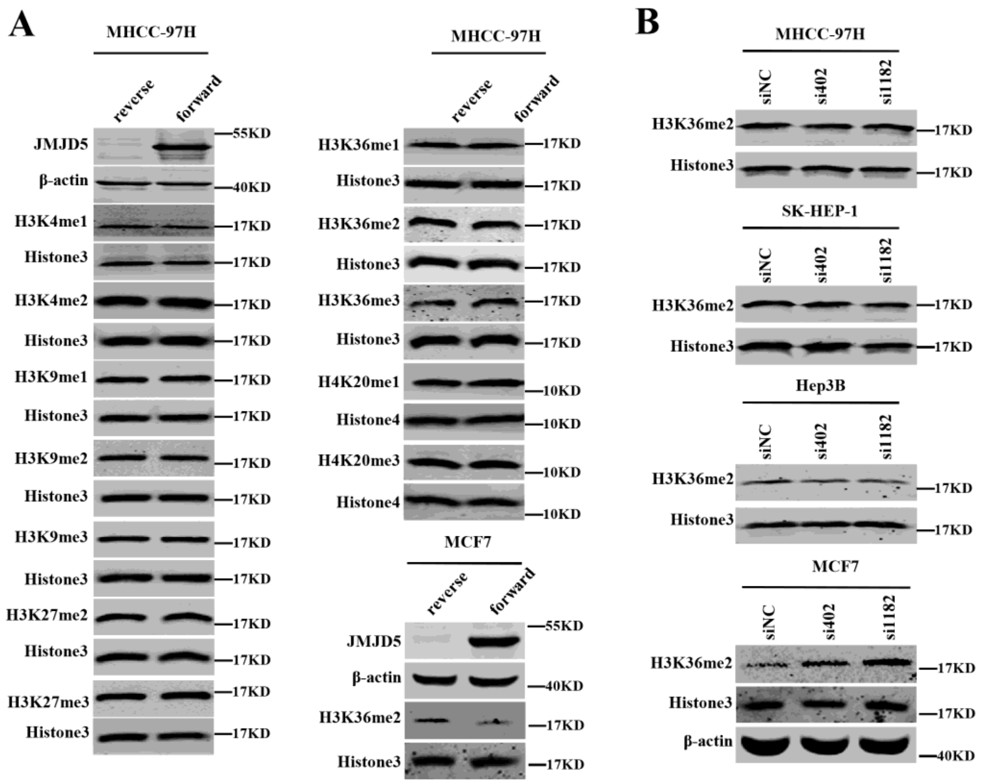

Supplemental Figure 7. The effects of *JMJD5* knockdown and overexpression on histone methylation. (A) Histone methylation was evaluated by western blot after ectopic JMJD5 expression (forward) in MHCC-97H and MCF7 cells; a vector containing the reverse *JMJD5* sequence (reverse) was used as a negative control, and both β-actin and histone H3 served as internal controls. **(B)** The H3K36me2 level was evaluated in MHCC-97H, SK-hep1, Hep3B HCC cells and MCF7 breast cancer cells after *JMJD5* knockdown with siRNAs (si402 and si1182), as shown by western blot. siNC was used as a negative control.

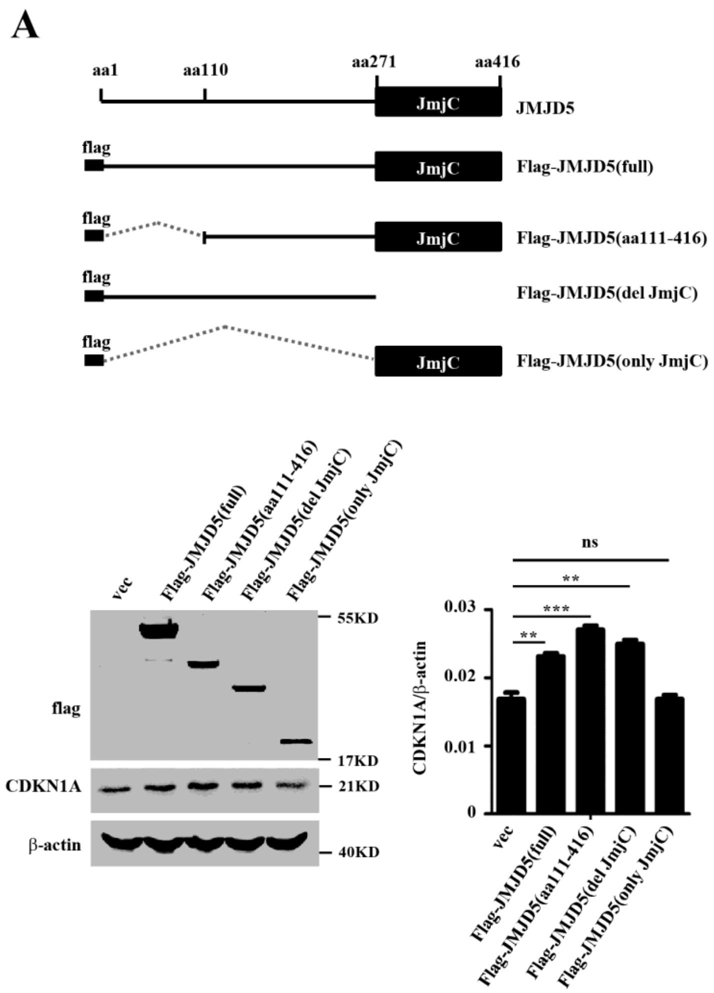

Supplemental Figure 8. The N-terminal of JMJD5 is necessary for CDKN1A activation.

(A) Three flag fused JMJD5 deletion mutants and flag fused full length JMJD5 were constructed, and westernblotting showed that CDKN1A is activated by the N-terminal but not the JmjC domain of JMJD5. Flag antibody was used to detect the expression of all mutants, β-actin was served as internal control. The bands' intensity of CDKN1A and β-actin was measured and the value of CDKN1A/β-actin was calculated. The experiment was repeated three times, two-tailed t-tests were used to calculate significant differences. **, P<0.01; ***, P < 0.001; ns, no significant difference.

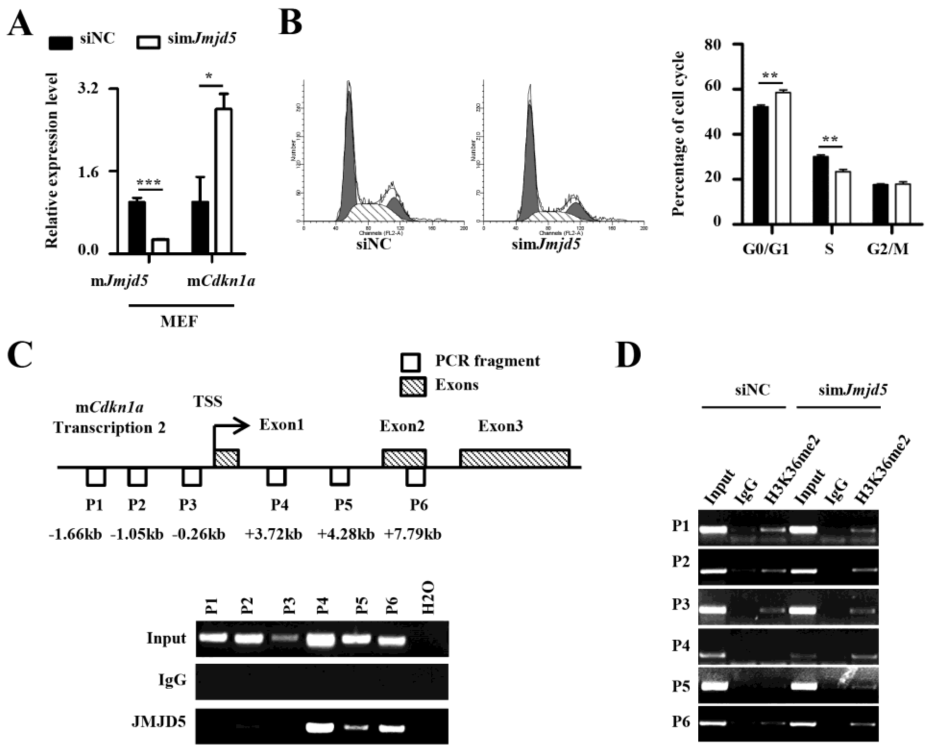

Supplemental Figure 9. Jmjd5 negatively regulates *Cdkn1a* transcription through H3K36me2 demethylase in MEF cell. (A) MEF cells were transfected with siRNA against mouse *Jmjd5*, after 48 hours, RNA was collected and the expression of *Jmjd5* and *Cdkn1a* were evaluated by real-time PCR. **(B)** The cell cycle was tested after *Jmjd5* knockdown (left), and the percentage of each cell cycle phases were statistically calculated (right). **(C)** Anti-Jmjd5 antibody was used to conduct ChIP assay, and the distribution of Jmjd5 on *Cdkn1a* gene locus was detected by semi-quantitative PCR. The blank boxes represented the primers' position, the shadowy boxes indicated the exons' position on the second transcript variant (Transcription 2) and the numbers showed the distance from primer to transcription start site (TSS). H_2O and IgG were used as negative control. **(D)** Anti-H3K36me2

antibody was used to carry out ChIP assay, and the alteration of this histone modification on *Cdkn1a* gene locus after *Jmjd5* knockdown was evaluated by semi-quantitative PCR. Two-tailed t-tests were used to calculate significant differences. *, P< 0.05; **, P<0.01; ***, P < 0.001.

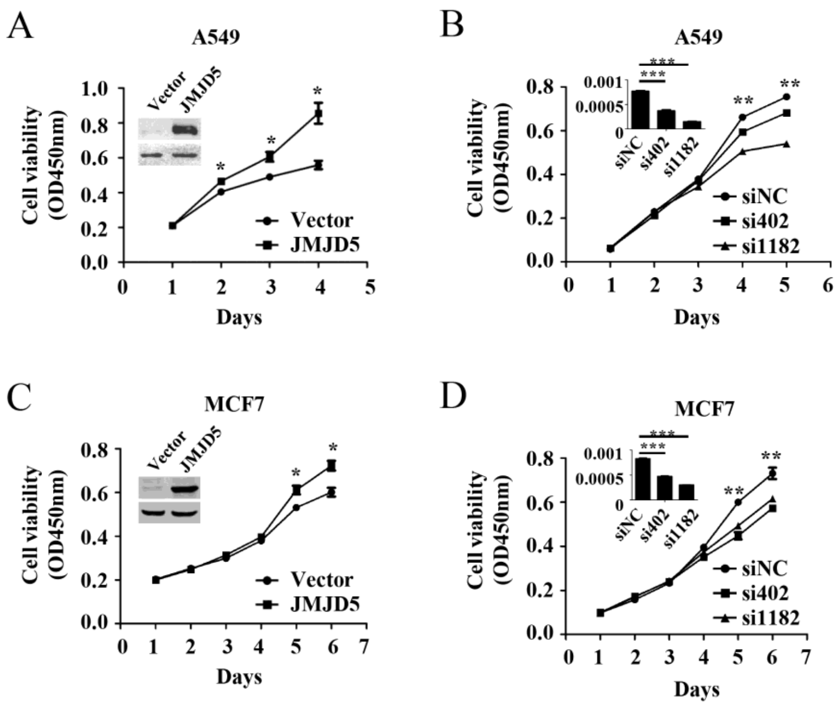

Supplemental Figure 10. JMJD5 inhibits the proliferation of A549 lung cancer cells and MCF7 breast cancer cells. (A-D) A549 lung cancer cells and MCF7 breast cancer cells were transfected with plasmid expressing JMJD5 or siRNAs against *JMJD5* (si402 and si1182), and cell viability was measured every day to delineate the cell growth curve. siNC and empty vector were served as a negative control. The expression of JMJD5 was measured by western blotting or real-time PCR and showed on the upper left of each figures. All experiments were repeated at least three times, and two-tailed *t*-tests were used to calculate significant differences. *, $P < 0.05$; **, $P < 0.01$.

Real-time PCR primers utilized in this study

Genes	Sequences
JMJD5	Forward: CTGTCCCAGTGGAAGTTGGT<br>Reverse: GTAGTCGGGGATGCTGATGT
β-actin	Forward: AGAGCCTCGCCTTTGCCGATCC<br>Reverse: CTGGGCCTCGTCGCCCACATA
CDK1	Forward: AAACTACAGGTCAAGTGGTAGCC<br>Reverse: TCCTGCATAAGCACATCCTGA
CDK2	Forward: CCAGGAGTTACTTCTATGCCTGA<br>Reverse: TTCATCCAGGGGAGGTACAAC
CDK4	Forward: ATGGCTACCTCTCGATATGAGC<br>Reverse: CATTGGGGACTCTCACACTCT
CDK5	Forward: GGAAGGCACCTACGGAACTG<br>Reverse: GGCACACCCTCATCATCGT
CDK6	Forward: GCTGACCAGCAGTACGAATG<br>Reverse: GCACACATCAAACAACCTGACC
CDK7	Forward: TGTATGGTGTAGGTGTGGACA<br>Reverse: TGCAAAGGTATTCCAGGGAAAC
CDK8	Forward: ACCTGTTTGAATACGAGGGCT<br>Reverse: TGCCGACATAGAGATCCCAGT
CDK9	Forward: ATGGCAAAGCAGTACGACTCG<br>Reverse: GCAAGGCTGTAATGGGGAAC
CDK10	Forward: GCCTGCGTCATCCGAACAT<br>Reverse: AGGGTGTTGGCATATTCTCCA
CDKN1A	Forward: CGATGGAACTTCGACTTTGTCA<br>Reverse: GCACAAGGGTACAAGACAGTG
CDKN1B	Forward: AACGTGCGAGTGTCTAACGG<br>Reverse: CCCTCTAGGGGTTTGTGATTCT
CDKN1C	Forward: GCGGCGATCAAGAAGCTGT<br>Reverse: GCTTGGCGAAGAAATCGGAGA
CDKN2A	Forward: ATGGAGCCTTCGGCTGACT<br>Reverse: GTAACTATTCGGTGCGTTGGG
CDKN2C	Forward: GGGGACCTAGAGCAACTTACT<br>Reverse: CAGCGCAGTCCTTCCAAAT
CDKN2D	Forward: AGTCCAGTCCATGACGCAG<br>Reverse: ATCAGGCACGTTGACATCAGC
RB1	Forward: CACTTTGTGAACGCCTTCTGT<br>Reverse: CACGTTTGAATGTCTCCTGAACA
RBL1	Forward: CTGGACGACTTTACTGCCATC<br>Reverse: TCCAACCGTGGGAATAATGCT
RBL2	Forward: GGAGGAAATTGGGACTCTCTCA<br>Reverse: AGACGACTCAAGCTATGCGTA

CCNA1	Forward: GAGGTCCCGATGCTTGTCAG<br>Reverse: GTTAGCAGCCCTAGCACTGTC
CCNA2	Forward: GGATGGTAGTTTTGAGTCACCAC<br>Reverse: CACGAGGATAGCTCTCATACTGT
CCNB1	Forward: TTGGGGACATTGGTAACAAAGTC<br>Reverse: ATAGGCTCAGGCGAAAGTTTTT
CCNB2	Forward: TTGGCTGGTACAAGTCCACTC<br>Reverse: TGGGAACTGGTATAAGCATTGTC
CCNC	Forward: CCTTGCATGGAGGATAGTGAATG<br>Reverse: AAGGAGGATACAGTAGGCAAAGA
CCND1	Forward: GCTGCGAAGTGGAAACCATC<br>Reverse: CCTCCTTCTGCACACATTTGAA
CCND2	Forward: ACCTTCCGCAGTGCTCCTA<br>Reverse: CCCAGCCAAGAAACGGTCC
CCND3	Forward: TACCCGCCATCCATGATCG<br>Reverse: AGGCAGTCCACTTCAGTGC
CCNE1	Forward: ACTCAACGTGCAAGCCTCG<br>Reverse: GCTCAAGAAAGTGCTGATCCC
CCNE2	Forward: GGAACCACAGATGAGGTCCAT<br>Reverse: CCATCAGTGACGTAAGCAAACT
CCNF	Forward: CCCCGAAGATGTGCTCTTTCA<br>Reverse: GCCTTCATTGTAGAGGTAGGCT
TP53	Forward: CAGCACATGACGGAGGTTGT<br>Reverse: TCATCCAAATACTCCACACGC
Pre-*CDKN1A*	Forward: GACACTCCATAATACCCCTC<br>Reverse: CTGAGACTAAGGCAGAAGATG
Pre-*GAPDH*	Forward: GGACTGGCTTTCCCATAATTT<br>Reverse: GTTCAGCTCAGGGATGACCTT
mCdkn1a	Forward: CCTGGTGATGTCCGACCTG<br>Reverse: CCATGAGCGCATCGCAATC
mJmjd5	Forward: CTGGTCCAGGTGCTAGGAAG<br>Reverse: AGAATGCAGGACAGGAATGG
mGapdh	Forward: AAATGGTGAA GGTCGGTGTG<br>Reverse: TGAAGGGGTCGTTGATGG

ChIP-PCR primers

Names	Sequences
h*CDKN1A*-P1	Forward: CTGAGGGGAGGCTCATACTG<br>Reverse: CAGAGCCAGGATGAATTGGT
h*CDKN1A*-P2	Forward: CACCTTTCACCATTCCCCTA<br>Reverse: CCCTTCCTCACCTGAAAACA
h*CDKN1A*-P3	Forward: GAAATGCCTGAAAGCAGAGG<br>Reverse: GATTGTGGCTAAACCCCAGA

h*CDKN1A*-P4	Forward: GAGGTCAGGGGTGTGAGGTA<br>Reverse: GGGCTCAGAGAAGTCTGGTG
h*CDKN1A*-P5	Forward: CTCTCCAATTCCCTCCTTCC<br>Reverse: AAGCACCTGGAGCACCTAGA
h*CDKN1A*-P6	Forward: TGCCGAAGTCAGTTCCTTGT<br>Reverse: CTGTGAACGCAGCACACAC
h*CDKN1A*-P7	Forward: TCTAACCAGCCTGTCAGTCG<br>Reverse: CCCACCCCTCAAAGACATGA
h*CDKN1A*-P8	Forward: GGCTGAAGTCTAAGGCAGGA<br>Reverse: AGGCACCCTCTCCTCTTCTA
h*CDKN1A*-P9	Forward: ATGATTCTCCCACCTCTGCC<br>Reverse: CCTGAGTCCTGTGCTGTAGT
h*CDKN1A*-P10	Forward: GCTGCCAGGTGTCTAGACTT<br>Reverse: CCTGCCAACTACCTCCTTCA
h*CDKN1A*-P11	Forward: TGGTAGGAAGACGTCACCTG<br>Reverse: CCATTAGCGCATCACAGTCG
h*CDKN1A*-P12	Forward: CGATGGAACTTCGACTTTGTCA<br>Reverse: GCACAAGGGTACAAGACAGTG
h*CDKN1A*-P13	Forward: ACCAGGCTCAGAGAGGTAGA<br>Reverse: CCCATCCCTGCAGAGTTACT
h*CDKN1A*-P14	Forward: TCATGTACATACCCTGGCCG<br>Reverse: AAGGAGAACACGGGATGAGG
h*CDKN1A*-P15	Forward: AATGGACTGGAAGGGGAAGG<br>Reverse: GATGGGGTGGATGAGGAAGG
h*CDKN1A*-P16	Forward: AACCACCCCAAGACTGAGTG<br>Reverse: TGTGAAGTCCCTGGAAGTCC
h*JMJD5*-P1	Forward: ACCTGAGGCCAGCCTATTCT<br>Reverse: CAGGACACAGACCCTCCAAT
h*JMJD5*-P2	Forward: TCCACAACACAAAAGGGTGA<br>Reverse: GTTTGAACTCAGCAGCCACA
h*JMJD5*-P3	Forward: AGCGGTTTCCCTTTTCACTT<br>Reverse: GATCATGCTCATCCCCATTT
h*JMJD5*-P4	Forward: GACCCCAAGCTCAGTACGAA<br>Reverse: GACACACTCCTGCCACCTTT
h*JMJD5*-P5	Forward: CTCGTCTCTTGGCAGTGGTT<br>Reverse: CTAAAAGCAGCGGACACACA
m*Cdkn1a*-P1	Forward: ACACACGACCTAGACAGCAA<br>Reverse: GCAGGTTTCATGGGTTGGAG
m*Cdkn1a*-P2	Forward: AGCTGAGGGACCCATGAAAA<br>Reverse: GTCACAAACCAGCACTGAGG
m*Cdkn1a*-P3	Forward: TTGGGAGTTTGTGTGGAGGT<br>Reverse: AGGAAGAGGAAGTGTGGACG
m*Cdkn1a*-P4	Forward: GCACTGGATTGAGACCAGAATC<br>Reverse: CCAAATAGGTCACTGTGCCG

m*Cdkn1a*-P5	Forward: GTTTCAGAGAGGACACTCAGGC<br>Reverse: CTTGATCTCCACGCCCAAAG
m*Cdkn1a*-P6	Forward: TACTTCCTCTGCCCTGCTGC<br>Reverse: TCAGACACCAGAGTGCAAGACAG

Sequences for siRNA and Primers for *CDKN1A* Promoter construction

siRNA	Sequences
siNC	5'-UUCUCCGAACGUGUCACGUTT-3'<br>5'-ACGUGACACGUUCGGAGAATT-3'
si402	5'-GGGACAUCCUUCUUAAAGUdtdt-3'<br>5'-ACUUUAAGAAGGAUGUCCCdtdt-3'
si1182	5'-CGGUGAAAUACUGGCAUUAdtdt-3'<br>5'-UAAUGCCAGUAUUUCACCGdtdt-3'
si777	5'-CAGCAAAUACAUCGUGAAUGAdtdt-3'<br>5'-UCAUUCACGAUGUAUUUGCUGAU-3'
siUTR	5' -CGCUGUCACUGAUCCCAAUUACUCUdtdt-3'<br>5'-AGAGUAAUUGGGAUCAGUGACAGCGGC-3'
si*CDKN1A*	5'-UCACUGUCUUGUACCCUUGUdtdt-3'<br>5'-ACAAGGGUACAAGACAGUGAdtdt-3'
sim*Jmjd5*	5'-CGAGACGCACAUUCUUCAUAAdtdt -3'<br>5'-UUAUGAAGAAUGUGCGUCUCGdtdt -3'
CDKN1A-Promoter-Full	Forward: GGGGTACCGGATCCCTGTAGAGATGCT<br>Reverse: CCGCTCGAGGATCCTAGACGAACTTACTC
CDKN1A -Promoter-T1	Forward: GGGGTACCCAGTTTCCCCAGCAGTG<br>Reverse: CCGCTCGAGGATCCTAGACGAACTTACTC
CDKN1A -Promoter-T2	Forward: GGGGTACCGCTCGGCGCTGGGCAGCC<br>Reverse: CCGCTCGAGGATCCTAGACGAACTTACTC

Primers for JMJD5 deletion mutants construction

Names	Sequences
Flag-JMJD5(full)	Forward:CGCGAATTCATGGATTACAAGGATGACGACGATAAG<br>GCTGGAGACACCCACTGCCC<br>Reverse: CGCCTCGAG CTACGACCACCAGAAGCTGAC
Flag-JMJD5(aa111-416)	Forward:CGCGAATTCATGGATTACAAGGATGACGACGATAAGGA<br>GGATGCCAACACTGTGGCC<br>Reverse: CGCCTCGAG CTACGACCACCAGAAGCTGAC
Flag-JMJD5(del JmjC)	Forward:CGCGAATTCATGGATTACAAGGATGACGACGATAAG<br>GCTGGAGACACCCACTGCCC<br>Reverse: CGCCTCGAG CTAGACGTCCCTTGGCTCATTCACG

Flag-JMJD5(only JmjC)	Forward:CGCGAATTCATGGATTACAAGGATGACGACGATAAG GGGTACCTTGCTCAGCACCAG<br>Reverse: CGCCTCGAG CTACGACCACCAGAAGCTGAC
